# HIV sero-status of healthcare workers in Addis Ababa public hospitals post exposure to infected blood and body fluids: A cross-sectional study, October 2022

**DOI:** 10.1017/S0950268823000754

**Published:** 2023-05-23

**Authors:** Ousman Adal Tegegne, Asmamaw Abebe Adissie

**Affiliations:** 1 Department of Emergency, Bahir Dar University College of Medicine and Health Sciences, Bahir Dar, Ethiopia; 2 Department of Emergency, Addis Ababa University College of Medicine and Health Sciences, Addis Ababa, Ethiopia

**Keywords:** Blood and body fluids, emergency, Ethiopia, HIV, post exposure

## Abstract

The study investigated the sero-status of human immunodeficiency virus among healthcare workers in Addis Ababa public hospitals. A multi-centered, institutional-based, cross-sectional study was conducted from 18 September 2022 to 30 October 2022. A simple random sampling method and a semi-structured, self-administered questionnaire were used to collect the data, which were analyzed using the Statistical Package for Social Sciences (SPSS) version 25. A binary logistic regression model was used to identify the factors associated with the human immunodeficiency virus sero-status of healthcare workers post exposure to infected blood and body fluids. Of the 420 study participants who were exposed to blood and body fluids, 403 (96%) were non-reactive. Healthcare workers who had 20–29 years of work experience had approximately six times higher odds of testing positive for the human immunodeficiency virus (AOR = 6.21, 95% CI: 2.39, 9.55). Healthcare workers who did not use personal protective equipment properly had five times higher odds of testing positive for the human immunodeficiency virus (AOR = 5.02, CI: 3.73, 9.51). This study showed that, among those healthcare workers who tested positive for the human immunodeficiency virus infection, the majority were from the emergency department. Healthcare workers who did not use personal protective equipment properly had higher odds of testing positive for the human immunodeficiency virus.

## Introduction

In every clinical setting, healthcare workers (HCWs) often face numerous occupational hazards, such as being exposed to the human immunodeficiency virus (HIV), because they are in continuous direct contact with their patients [1] to provide them with immediate care. Because of high patient volumes in the emergency department, nurses there are overloaded, and hence they are at the highest risk of such exposure [2].

Recent research shows that exposure to HIV is mostly caused by sexual intercourse (62.6%), and developing countries account for more than 90% of these events [3]. However, among HCWs, this can sometimes be caused by exposure to the blood and body fluids (BBFs) of infected patients [2]. Every year, approximately 15 000 healthcare workers are infected with this preventable infection [4, 5].

The consequences of BBF exposure include not only the risk of HIV transmission but also the transmission of various pathogens such as hepatitis B virus (HBV), hepatitis C virus (HCV), and other blood-borne pathogens that result in systemic and localised site infection [6, 7]. Furthermore, the fear of infection might lead to significant anxiety and depression issues [8, 9].

In a study conducted among the HCWs in Iran, the highest rates of exposure were found to be among young and recently employed nurses with less than 3 years of experience (74.6%). Of those HCWs, 3% of them had been infected by known HIV-positive patients, 13% were known hepatitis B serum antigen (HBs-Ag) positive, and 2% were HCV-positive patients [10].

The frequency and annual rate of HIV prevalence are higher among HCWs in the emergency department. For instance, according to a research conducted by Gourni P et al., of the exposed HCWs, 22.3% were found to be HIV positive [2, 9].

A systematic review and meta-analysis conducted in 2017 in 21 African countries showed that, of 65.5% of exposed HCWs, 25% tested positive for the human immunodeficiency virus [11]. Higher patient flow, a lower ratio of HCWs to patients, failure to implement standard precautions, inadequate supply of basic safety equipment, lack of training, and inadequate supply of personal protective equipment (PPE) are factors that contribute to the higher prevalence of infection [11, 12].

Currently, in Ethiopia, there is a lack of reports that quantify the pooled prevalence of HIV among HCWs. Moreover, the epidemiology of HIV in Ethiopia has been on the rise and has been dynamically changing over the past two decades, along with poor compliance with standard precautions among HCWs [13–15].

## Methods

### Study design and setting

A multi-centered, institutional-based, cross-sectional study was conducted among HCWs in public hospitals in Addis Ababa city from 18 September 2022 to 30 October 2022. Addis Ababa is the capital city of Ethiopia, which is located in the central part of the country. Additionally, it is the seat of the African Union and the United Nations’ World Economic Commission for Africa. Here, there are more than 53 hospitals, of which 13 are public hospitals and more than 40 are private hospitals [16–18]. The study population contained all HCWs who were working in five randomly selected public hospitals in Addis Ababa city.

### Sample size, sampling procedure, and technique

The actual sample size for the study was determined using a single population proportion formula *n* = [(*zα*/2)^2^*p*(1−*p*)]/*d* ^2^}, where *n* = sample size, *zα*/2 = 95% confidence level, *p* = the proportion of HIV-positive HCWs among those exposed to BBFs in the previous study (48.2%) [19], and *d* = margin of error (0.05). By considering 10% of non-response rate, the final sample size of the study was 422. To determine the representativeness of the sample, by using the lottery method, the principal investigators were randomly selected from two-thirds of the total hospitals (three from five). The sample size for each hospital was proportionally allocated based on the number of HCWs in each hospital. Individuals who fulfilled the inclusion criteria were selected using systematic random sampling at two intervals from their list. Their lists were obtained from the office of the chief clinical director of each hospital. Then, consent was obtained from each study participant.

### Inclusion criteria

All HCWs who were exposed to BBFs over the past 12 months and tested positive for HIV after exposure using a rapid diagnostic test (HIV serum-antibody test kit) were included.

### Exclusion criteria

Healthcare workers who did not know their HIV sero-status, meaning they had not been exposed or tested after exposure in the previous 12 months, were excluded.

### Operational definitions

HIV prevalence greater than 1.2% or said to be high prevalence [20].

### Data collection tools

The English version of the self-administered questionnaire was used to collect the data. The tools were divided into two sections: participant sociodemographic information and HIV sero-status data from a modified version of the study conducted in Cape Town, South Africa [21]. The supervisors were given the questionnaire for each study participant. Then, the participants themselves filled out the questionnaire, as directed by the supervisors and the principal investigators.

### Data quality control

Training was provided to supervisors, and appropriate supervision was provided. A pre-test was conducted 2 weeks before the actual data collection using 5% of the sample size. The internal consistency of instruments in the pre-test data (questionnaire) was confirmed (Cronbach’s alpha = 0.86). Two professional experts (one from the English language and one from medicine) validated the tool. After the pre-test, some explanations were modified and re-edited. The collected data were checked for completeness, and some unclear statements were corrected.

### Patient and public Involvement

No patients were involved in this study.

### Data processing and analysis

Before analyzing the data, it was cleaned up and cross-checked. The data were entered into Epi Data version 4.6.0.4 and exported to SPSS version 26 for further analysis. A binary logistic regression model was used to estimate the associated factors of all independent variables with a *p*-value of <0.05. The model fitness of the variable was tested using the Hosmer–Lemeshow test. All independent variables with a *p*-value of <0.05 from a bivariate logistic regression analysis were considered for fitting into a multivariable logistic regression analysis to control for the possible effect of confounders. Descriptive statistics such as percentage, mean, median, and standard deviation were used. Tables, graphs, and narrations were used for data presentation.

## Results

### Sociodemographic characteristics of study participants

Of the 420 HCWs who participated in the study, which had a response rate of 99.5%, most of the 236 HCWs (56%) were female. Two hundred and fifty-five (62%) HCWs were between the ages of 20 and 29, with a mean age of 30.18 ± 4.37. Most study participants, 301 (72%), were nurses, and 278 (66%) had 1–9 years of work experience. More than half of the HCWs (246, or 58.5%) used personal protective equipment correctly (Table 1).

**Table 1.** Sociodemographic characteristics of study participants, October 2022

Variables		No. (%), *N* = 420
Sex	Male	185(44)
	Female	235 (56)
Age	20–29	255 (62)
	30–39	130 (31)
	40–49	35 (7)
Educational status	physician	72 (17)
	Nurse	301 (72)
	Midwifery	47 (11)
Work experience	1–9 years	278 (66)
	10–19 years	121 (29)
	20–29 years	21 (5)
Working institution	Tikur Anbessa Specialized Hospital	117 (28)
	St. Paulo’s Specialized Hospital	124 (30)
	Alert Specialized Hospital	65 (15)
	Yekatit 12 Hospital	65 (15)
	Zewditu Memorial Hospital	49 (12)
Proper use of personal protective equipment	Yes	246 (58.5)
	No	174 (41.5)

### Human immune deficiency virus sero-status of healthcare workers

Of the 420 study participants who were exposed to blood and body fluids over the past 12 months, 403 (96%) were non-reactive, while 17 (4%) were reactive.

### HIV sero-status of HCWs with their sociodemographic characteristics

In a chi-square test analysis with a p-value of <0.05, factors such as the proper use of personal protective equipment, educational status, and work experience were significantly associated with the post-exposure HIV sero-status of healthcare workers (Table 2).

**Table 2.** HIV sero-status of HCWs with their sociodemographic characteristics

Sex	*N* = 420	Non-reactive (*n* = 403)	Reactive (*n* = 17)	*X* ^2^	*p*-value
Age	Male	178 (96.2)	7(3.8)	2.343	0.123
	Female	225 (95.7)	10 (4.3)		
Educational status	20–29	246 (96.5)	9 (3.5)	1.134	0.152
	30–39	125 (96)	5 (4)		
	40–49	32 (91.4)	3 (8.6)		
Work experience	Physician	71 (98.6)	1 (1.4)	3.162	0.042*
	Nurse	289 (96)	12 (4)		
	Midwifery	43 (91.5)	4 (8.5)		
Proper use of personal protective equipment	1–9 years	266 (95.7)	12 (4.3)	1.657	0.012*
	10–19 years	117(96.7)	4 (3.3)		
	20–29 years	2 0 (95.2)	1 (4.8)		
	Yes	245 (99.6)	1 (0.4)	3.345	0.001*
	No	158 (90.8)	16 (9.2)		

* Significant at *p*-value <0.05.

### Factors associated with the post-exposure HIV sero-status of healthcare workers

The goodness of fit of the variable using the Hosmer–Lemeshow test displayed that the dependent variable was explained by the independent variables by 89.6%. In a binary logistic regression analysis with a *p*-value of <0.05, factors such as the proper use of personal protective equipment, educational status, and work experience were significantly associated with the post-exposure HIV sero-status of healthcare workers. Healthcare workers who had 20–29 years of work experience had approximately six times higher odds of testing positive for HIV (AOR = 6.21, 95% CI: 2.39, 9.55). HCWs who were not using PPE properly had approximately five times more odds of testing positive for HIV (AOR = 5.02, 95% CI: 3.73, 9.51). HCWs who were midwives had approximately four times higher odds of testing positive for HIV (AOR = 4.2, 95% CI: 3.17, 8.21) (Table 3).

**Table 3.** Factors associated with the HIV sero-status of healthcare workers, November 2022

	HIV sero-status of healthcare workers			
Variables	*NR*	*R*	COR (95% CI)	AOR (95% CI)
Sex				
Male	178	7	1.00	1.00
Female	225	10	0.32 (0.61, 4.3)	13.8 (0.45, 25.12)
Age				
20**–**29	246	9	1.00	1.00
30**–**39	125	5	0.75 (0.34, 2.43)	4.23 (0.71, 14.05)
40**–**49	32	3	3.40 (0.29, 64)	4.57 (0.19, 2.51)
Educational status				
Physician	71	1	1.00	1.00
Nurse	289	12	0.17 (0.25, 3.08)	4.8 (0.24, 4.21)
Midwifery	43	4	3.10 (1.12, 6.23)*	4.2 (3.17, 8.21)*
Work experience				
1**–**9 years	266	12	1.00	1.00
10**–**19 years	117	4	4.73 (2.15, 9.71)	3.11 (0.26, 7.34)
20**–**29 years	20	1	6.75 (0.57, 9.267)*	6.21 (2.39, 9.55)*
Proper use of personal protective equipment				
Yes	245	1	1.00	1.00
No	158	16	5.70 (3.25, 8.00)*	5.02 (3.73, 9.51)*

*Note.* 1:00: reference.

AOR, adjusted odd ratio; OR, crude odd ratio; NR, non-reactive; R, reactive.

* *P*-value < 0.005.

## Discussion

This study found that the prevalence of HIV among HCWs’ post exposure to infected BBFs over the past 12 months was 17 (4%). This means that, although various technologies and prevention methods have been developed, HCWs are still at risk of contracting HIV in their workplaces. This is comparable with the study that was reported from South Africa (4%) [21], Tanzania (3%) [22], and Nigeria (4.2%) [23].

However, the result is much higher than the studies conducted in Iran (1.7%) [24], Australia (2.1%) [25], Kenya (1.5%) [26], and Tunisia (1.7%) [27]. This discrepancy may be due to variation among the study participants, a lack of PPE, a higher patient load, and the infrequent use of PPE among participants in this study setting. For instance, in this study, HCWs infrequently used PPE due to its shortage in the COVID era [24–26]. Additionally, there is a higher patient flow along with greater consumption of PPE in this study setting and in the country as a whole, which subsequently can lead to higher exposure to HIV.

In this study, healthcare workers who had 20–29 years of work experience had six times higher odds of testing positive for HIV (AOR = 6.21, 95% CI: 2.39, 9.55). HCWs who were not using PPE properly had five times more odds of testing positive for HIV (AOR = 5.02, 95% CI: 3.73, 9.51). HCWs who were midwives had four times higher odds of testing positive for HIV (AOR = 4.2, 95% CI: 3.17, 8.21). This is supported by many studies, such as those in Tunisia [27], South Africa [21], and Kenya [26]. The reason could be described as the availability of PPE in healthcare facilities, influencing HCWs’ habits of using PPE during patient care and procedures, thereby reducing exposure to HIV and its impact on the outcome of exposure. Furthermore, the infrequent availability of PPE reduced HCWs’ compliance to wearing PPE such as gloves, face masks, face shields, and aprons, potentially amplifying their exposure to HIV infection; and finally, increasing the transmission of blood-borne pathogens. Caring for patients without the use of proper PPE can also increase the risk of HIV infection due to exposure to contaminated BBFs and can lead to anxiety and further exposure to HIV infection [13, 28].

### Implications of the study

This study will be used to provide information to healthcare providers, non-governmental organisations, and policymakers for appropriate planning and interventions regarding HCWs’ exposure to HIV infection.

This study also provides new knowledge regarding occupational exposure to HIV infection among healthcare workers. Moreover, the results of this study serve as baseline data for further longitudinal and action-based studies.

### Strength and limitations

As its strength, this study was conducted in five randomly selected public hospitals; thus, it could be generalised to all HCWs working in public hospitals. The data was collected using a self-administered questionnaire given to each participant; hence, it is susceptible to recall bias.

## Conclusion

This study showed that a higher proportion of healthcare workers at the emergency department tested positive for the human immunodeficiency virus infection among those who were exposed to blood and body fluids and had been tested immediately. Healthcare workers who did not use personal protective equipment properly had higher odds of testing positive for the human imm/unodeficiency virus.

## Data Availability

The data that support the findings of this study are available upon reasonable request from the corresponding author.

## Abbreviations

BBFs: Blood and body fluids
ED: Emergency departments
HBV: Hepatitis B virus
HCV: Hepatitis C virus
HCWs: Healthcare workers
PPE: Personal protective equipment
SPSS: Statistical Packages for Social Sciences

## References

[1] Simieneh A, Tadesse M, Kebede W, Gashaw M and Abebe G (2022) Combination of Xpert® MTB/RIF and DetermineTM TB-LAM Ag improves the diagnosis of extrapulmonary tuberculosis at Jimma University Medical Center, Oromia, Ethiopia. PloS One 17(2), e0263172. 35113917 10.1371/journal.pone.0263172 PMC8812938

[2] Mengistu DA, Dirirsa G, Mati E, Ayele DM, Bayu K, Deriba W, Alemu FK, Demmu YM, Asefa YA and Geremew A (2022) Global occupational exposure to blood and body fluids among healthcare workers: Systematic review and meta-analysis. Canadian Journal of Infectious Diseases and Medical Microbiology 2022, 5732046. 35692264 10.1155/2022/5732046 PMC9187485

[3] Gebremariyam BS, et al. (2019) Determinants of occupational exposure to blood and body fluids, healthcare workers’ risk perceptions and standard precautionary practices: A hospital-based study in Addis Ababa, Ethiopia. Ethiopian Journal of Health Development 33(1), 4–11.

[4] British Columbia Centre for Disease Control (2017) Blood and body fluid management. Section 6-20, p. 7.

[5] Zhang L, Li Q, Guan L, Fan L, Li Y, Zhang Z and Yuan S (2022) Prevalence and influence factors of occupational exposure to blood and body fluids in registered Chinese nurses: A national cross-sectional study. BMC Nursing 21(1), 1–13. 36333812 10.1186/s12912-022-01090-y PMC9636689

[6] Abere G, Yenealem DG and Wami SD (2020) Occupational exposure to blood and body fluids among health care workers in Gondar Town, Northwest Ethiopia: A result from cross-sectional study. Journal of Environmental and Public Health. 2020, 3640247. 10.1155/2020/3640247. 32508935 PMC7245691

[7] Madiba TK, Nkambule NR, Kungoane T and Bhayat A (2018) Knowledge and practices related to hepatitis B infection among dental and oral hygiene students at a University in Pretoria. Journal of International Society of Preventive & Community 8(3), 200–204. 10.4103/jispcd.JISPCD_31_18. PMC5985674 29911055

[8] Yenesew MA and Fekadu GA (2014) Occupational exposure to blood and body fluids among health care professionals in Bahir Dar town, Northwest Ethiopia. Safety and Health at Work 5(1), 17–22. 24932415 10.1016/j.shaw.2013.11.003 PMC4048007

[9] Yasin J, Fisseha R, Mekonnen F and Yirdaw K (2019) Occupational exposure to blood and body fluids and associated factors among health care workers at the University of Gondar Hospital, Northwest Ethiopia. Environmental Health and Preventive Medicine 24(1), 1–9. 30851726 10.1186/s12199-019-0769-9 PMC6408855

[10] Naderi H, Sheybani F, Bojdi A, Mostafavi I and Khosravi N (2012) Occupational exposure to blood and other body fluids among health care workers at a university hospital in Iran. Workplace Health & Safety 60(10), 419–422. 23054163 10.1177/216507991206001003

[11] Auta A, Adewuyi EO, Tor-Anyiin A, Aziz D, Ogbole E, Ogbonna BO and Adeloye D (2017) Health-care workers’ occupational exposures to body fluids in 21 countries in Africa: Systematic review and meta-analysis. Bulletin of the World Health Organization 95(12), 831. 29200524 10.2471/BLT.17.195735 PMC5710084

[12] Chalya PL, Seni J, Mushi MF, Mirambo MM, Jaka H, Rambau PF, Mabula JB, Kapesa A, Ngallaba SE, Massinde AN and Kalluvya SE (2015) Needle-stick injuries and splash exposures among health-care workers at a tertiary care hospital in North-Western Tanzania. Tanzania Journal of Health Research 17(2).

[13] Sahiledengle B, Tekalegn Y, Woldeyohannes D and Quisido BJE (2020) Occupational exposures to blood and body fluids among healthcare workers in Ethiopia: A systematic review and meta-analysis. Environmental Health and Preventive Medicine 25(1), 58. 10.1186/s12199-020-00897-y. 33010808 PMC7533038

[14] Ivanova Reipold E, Fajardo E, Juma E, Bukusi D, Bermudez Aza E, Jamil MS, Johnson CC, Farquhar C, Easterbrook P and Monroe-Wise A (2022) Usability and acceptability of oral fluid hepatitis C self-testing among people who inject drugs in coastal Kenya: A cross-sectional pilot study. BMC Infectious Diseases 22(1), 1–11. 36109704 10.1186/s12879-022-07712-9 PMC9479404

[15] Akalu TY, Aynalem YA, Shiferaw WS, Merkeb Alamneh Y, Getnet A, Abebaw A, Atnaf A, Abate A, Tilahun M, Kassie B and Aschale Y (2022) National burden of intestinal parasitic infections and its determinants among people living with HIV/AIDS on anti-retroviral therapy in Ethiopia: A systematic review and meta-analysis. SAGE Open Medicine 10, 20503121221082447. 35284074 10.1177/20503121221082447 PMC8908390

[16] Sahile AT, et al. (2019) Level-of-nurses-job-satisfaction-and-associated-factors-working-in-public-hospitals-of-Addis-Ababa-Ethiopiai. EC Nursing and Health Care 2, 1–7.

[17] Adal O and Abebe A (2022) First aid knowledge and practice toward students with epileptic seizure among governmental high school teachers in Addis Ababa, Ethiopia: Cross-sectional study. Epilepsy & Behavior 134, 108767. 10.1016/j.yebeh.2022.108767. 35772344

[18] Adal O and Emishaw S (2023) Knowledge and attitude of healthcare workers toward advanced cardiac life support in Felege Hiwot Referral Hospital, Bahir Dar, Ethiopia, 2022. SAGE Open Medicine 11, 20503121221150101. 36685795 10.1177/20503121221150101 PMC9850119

[19] Atlaw WD (2013) Patterns of Occupational Exposure to patients’ Body Fluids among Health Care Workers in Tikuranbesa University Hospital. Ethiopia: Addis Ababa.

[20] Gelibo T, Lulseged S, Eshetu F, Abdella S, Melaku Z, Ajiboye S, Demissie M, Solmo C, Ahmed J, Getaneh Y, Kaydos-Daniels SC, Abate E and EPHIA Study Group (2022) Spatial distribution and determinants of HIV prevalence among adults in urban Ethiopia: Findings from the Ethiopia population-based HIV impact assessment survey (2017–2018). PLoS One 17(7), e0271221. 35819961 10.1371/journal.pone.0271221 PMC9491827

[21] Adeola AO and Forbes PB (2022) Antiretroviral drugs in African surface waters: Prevalence, analysis, and potential remediation. Environmental Toxicology and Chemistry 41(2), 247–262. 34033688 10.1002/etc.5127

[22] Eyong EM, Ngwe NY, Nfuksai CN, Niba LL and Jane-Francis A (2022) Prevalence of occupational exposure to HIV and factors associated with compliance with post-exposure prophylaxis among health workers of the Biyem-Assi, Buea, and Limbe health districts of Cameroon maternal and child health and AIDS. International Journal of Maternal and Child Health and AIDS 11(1), e557. 35959456 10.21106/ijma.557 PMC9359212

[23] Tekalign T, Awoke N, Eshetu K, Walle BG and Guta MT (2022) HIV/AIDS post-exposure prophylaxis knowledge and uptake among health professionals in Africa: Systematic review and meta-analysis. HIV Medicine 23, 811–824. 10.1101/2022.02.03.22270379. 35355388

[24] Levaillant M, Lièvre G and Baert G (2019) Ending diabetes in Mexico. The Lancet 394(10197), 467–468. 10.1016/S0140-6736(19)31662-9 31402023

[25] Ifeoma A, Apalata T, Aviwe B, Oladimeji O and Abaver DT (2022) Prevalence of intestinal parasites in HIV/AIDS-infected patients attending clinics in selected areas of the eastern cape. Microbiology Research 13(3), 574–583.

[26] Nagata JM, Miller JD, Cohen CR, Frongillo EA, Weke E, Burger R, Wekesa P, Sheira LA, Mocello AR, Otieno P, Butler LM, Bukusi EA, Weiser SD and Young SL (2022) Water insecurity is associated with lack of viral suppression and greater odds of AIDS-defining illnesses among adults with HIV in western Kenya. AIDS and Behavior 26(2), 549–555. 34373987 10.1007/s10461-021-03410-w PMC8813828

[27] Besbes A, Nasri W, Nafti R and Bennasrallah C (2022) Knowledge, attitudes and practices about HIV: A pilot study among Tunisian dentists. World Journal of Dentistry 13(2), 156.

[28] Mekonnin T, Tsegaye A, Berihun A, Kassachew H and Sileshi A (2018) Occupational exposure to blood and body fluids among health care workers in Mizan Tepi university teaching hospital, bench Maji zone, south West Ethiopia. Medical Safety & Global Health 7, 2.

